# Enhancing Data Quality and Remote Accessibility in Clinical Trials: A SeroSelectTB Case Study in Ethiopia, Tanzania, and South Africa

**DOI:** 10.1055/a-2873-5092

**Published:** 2026-05-22

**Authors:** Sasho Najdov, Jordancho Arsov, Jovan Davchev, Tamirat Assefa, Jemrath Bikombo, Debora Kajeguka, Anna Okunola, Welile Nwamba, Aleksandar Josifoski, Carol Holm-Hansen

**Affiliations:** 1 Aether Dynamics GmbH, Vienna, Austria; 2 70605 Armauer Hansen Research Institute, Addis Ababa, Addis Ababa, Ethiopia; 3 108095 Kilimanjaro Christian Medical Centre, Moshi Urban, Kilimanjaro Region, Tanzania, United Republic of; 4 121470 Stellenbosch University Faculty of Medicine and Health Sciences, Cape Town, WC, South Africa; 5 25563 Norwegian Institute of Public Health, Oslo, Oslo, Norway

**Keywords:** clinical data management, data processing, data quality, clinical information systems, specific types, process management tools, clinical decision support, computer assisted decision-making

## Abstract

**Background:**

Ensuring data quality and maintaining remote accessibility remain key challenges in clinical trials conducted in low-resource settings. Although several software platforms and digital tools attempt to address these challenges, no single, comprehensive solution adequately meets the complex demands of decentralized and intermittently connected trial environments. Consequently, context-specific tools must be developed and adapted to ensure reliable performance under such constraints.

**Objective:**

The aim of this study was to enhance data quality and enable uninterrupted participant randomization in settings with limited internet and electricity through the implementation of an automated Quality Control (QC) reporting system and an online and offline participant randomization application.

**Methods:**

A centralized automated QC reporting system was developed using a Python-based script, which was integrated with the Research Electronic Data Capture system to extract and process data, and generate a web-based monitoring dashboard, which displayed data summaries, recruitment charts, and flags on missing or inconsistent records. Thereafter, a mobile randomization application built with React Native was developed for online and offline participant randomization, employing block randomization to ensure balanced allocation between study arms and enrollment continuity.

**Results:**

The automated QC system reduced data verification time from months to 1 hour, enabling real-time oversight and early error correction. The randomization application maintained balanced study arms and allowed uninterrupted participant enrollment during internet outages. These digital tools improved data completeness, reduced bias and human error, and increased trial efficiency across all study sites.

**Conclusion:**

Automation and offline functionality can significantly enhance data quality, efficiency, and integrity in clinical trials conducted in resource-constrained environments. The experience from this study demonstrates the value of combining automated QC and offline randomization tools to ensure robust and continuous study operations.

## Background and Significance

Clinical trials depend on strong data management and oversight systems. Current trial management commonly relies on electronic data capture (EDC) systems, centralized monitoring, and predefined quality control (QC) procedures. However, these systems often assume stable internet access, consistent power supply, and uniform regulatory environments, which are not guaranteed in low-resource settings and multinational studies. These limitations are particularly pronounced in trials conducted in high tuberculosis (TB)-burden countries (HBC), where infrastructure constraints intersect with urgent public health demands. Sites frequently manage connectivity gaps through delayed data entry, manual reconciliation, and retrospective monitoring, increasing operational burden and risk of data inconsistencies.

TB poses a significant global health challenge in HBC. 1 Early and accurate detection of active TB is crucial for prompt treatment initiation and effective disease control. To address this, the SeroSelectTB 2 3 4 study evaluated a rapid serological screening test designed for use at primary healthcare facilities in HBC settings. SeroSelectTB detects active pulmonary TB and supports the decision to refer presumptive TB patients for same-day confirmatory testing and start-of-treatment.

While an accurate and reliable TB triage test is essential for diagnostic algorithms, ensuring data quality and facilitating seamless trial operations are crucial in clinical research.

This paper highlights the importance of an automated QC reporting system and a hybrid offline/online mobile randomization application (app) in the context of the SeroSelectTB study conducted in Ethiopia, South Africa, and Tanzania. The Sero-SelectTB clinical trial and challenges faced are described in detail in a recent publication by Savic et al. 4 Because no system supports all data management dimensions in clinical trials, 5 these tools aim to fill gaps not supported by existing EDC systems. The QC system helps identify trends, data gaps, and entry errors, while the randomization app supports participant randomization in areas with limited internet connectivity or electricity.

This paper also addresses the functionality and benefits of the automated QC reporting system and the randomization app, showcasing their value in overcoming challenges faced in HBC settings, such as coordinating across multinational regulatory differences and managing intermittent connectivity. Ultimately, these tools contributed to the success of the SeroSelectTB study by ensuring data reliability and trial continuity.

## Challenges

### Multinational Study Coordination

Coordinating data management activities across three countries posed unique challenges that required careful planning and collaboration. Each country has national regulatory requirements, ethical committee rules and procedures, data privacy considerations, different electronic Case Report Forms (eCRFs) for data collection, language differences, and national/local conventions that could affect data entry and interpretation. This study measured diagnostic delay, and data consistency was extremely important because even the smallest error in the data points could distort the final results. To ensure consistency and standardization in data management practices across all study sites, it was essential to foster close collaboration and effective communication between the data management team and local stakeholders.

### Limited or No Access to Electricity and the Internet

In certain study locations, limited access to electricity and the internet posed challenges for data management operations. The availability and reliability of the power supply and the internet were essential for running electronic data collection, including the participant enrollment and data synchronization processes. Establishing effective offline data collection and synchronization methods was necessary to ensure data integrity and enable the seamless continuation of data management processes in these remote locations. Implementing strategies to mitigate the consequences of power outages and no internet access, and ensuring the integrity of data was a priority. Using mobile devices (tablets and smartphones) and ensuring synchronization with the central server once connectivity was restored required careful planning and technical expertise.

### Meeting Reporting Demands and Enabling Early Feedback

The study had obligatory participant enrollment and data quality deliverables. Meeting these demands while navigating different eCRFs, limited connectivity, and remote locations required efficient data collection, consolidation, and reporting processes. Providing accurate and timely updates was a priority to support effective decision-making and study management. Enabling early and accurate study updates would ensure early and quick error detection and correction. A key challenge addressed was obtaining data across sites and reporting missing information.

Overcoming these challenges necessitated proactive measures and innovative solutions. Collaborating with local stakeholders, implementing offline capabilities, and optimizing data collection and synchronization processes were the key strategies employed by the data management team.

## Methods

### Automatic Quality Control Report System

To enable study oversight across countries, address the differences in the eCRFs, and enable an early feedback mechanism, an automatic QC reporting system was developed. This system operates by extracting data from the eCRFs stored in REDCap (Research Electronic Data Capture). 6 The QC report system uses a Python 7 script that extracts the entered data on an hourly basis to initiate the QC process. The extracted data are then parsed and processed using custom algorithms and logic designed to identify specific issues, and compiled into an HTML web page using Jinja, 8 for easy viewing by healthcare workers and supervisors.

During the processing phase, the system thoroughly examines the data for missing values (e.g., unanswered required fields like treatment start dates), incorrect values (e.g., out-of-range entries such as impossible test scores or dates, or inconsistencies like a treatment date before enrollment), and participant identification errors (e.g., mismatched or duplicate IDs). It performs comprehensive checks to ensure data completeness and accuracy. Any inconsistencies or discrepancies detected are flagged and reported in the QC report. These reports provide healthcare workers with a clear overview of the data quality status, highlighting areas that require attention or correction.

The QC report system goes beyond error detection. It offers valuable insights and analytics for monitoring recruitment progress and comparing site performance. By aggregating and grouping randomizations, the system generates charts and statistics that enable supervisors to track recruitment numbers, identify trends, and spot irregularities or anomalies. This real-time oversight empowers supervisors to take swift action, providing guidance and support to ensure sites remain on track and recruitment efforts align with study objectives.

To ensure data is updated, the system includes a “Last Updated” timestamp, and reports refresh automatically after corrections in REDCap; no performance issues were observed with the REDCap API even as the database grew to thousands of records.

#### General Overview: Statistics of the Country

**Fig. 1:**
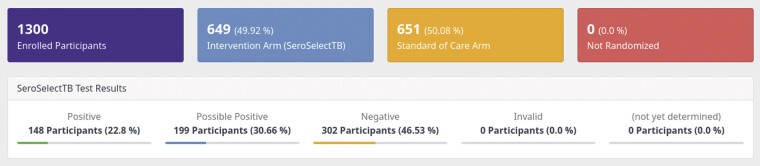
Study arm and SeroSelectTB result distribution dashboard. An overview of total enrolled participants, distribution across study arms, and SeroSelectTB test results, allowing quick assessment of balance and missing randomizations.

An overview of the statistics available at the top of the report is shown in Fig. 1 . These statistics include the total enrolled participants, the enrollment distribution across study arms, and the number of participants not randomized. Additionally, test results from the intervention arm (the SeroSelectTB results) are provided. This overview enables healthcare workers and supervisors to quickly assess any imbalance between the arms, participants not randomized, and SeroSelectTB test results.

#### Site Distribution and Trend Line

**Fig. 2:**
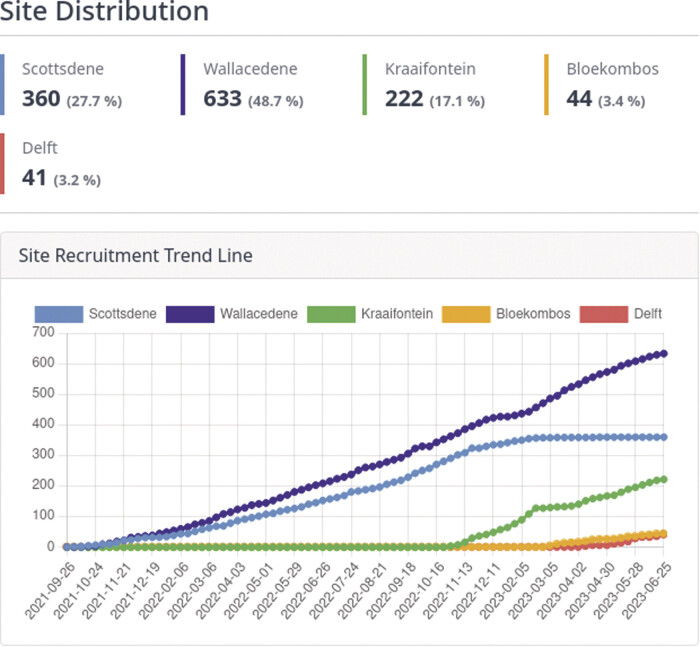
Site distribution chart showing participant enrollment over time. Displays participant distribution per site and monthly trend lines. A stalled recruitment pattern at Scottsdene in late 2022, early 2023, was detected and addressed.

Following the overview, the QC report shows participant distribution per site and a trend line on how each site has progressed through the months, as shown in Fig. 2 . This helps detect any sudden drop or stall in recruitment, which may indicate that a site is facing challenges that need to be addressed. For example, Fig. 2 indicates that the recruitment at Scottsdene stalled in early 2023.

#### Missing Data Statistics

**Fig. 3:**
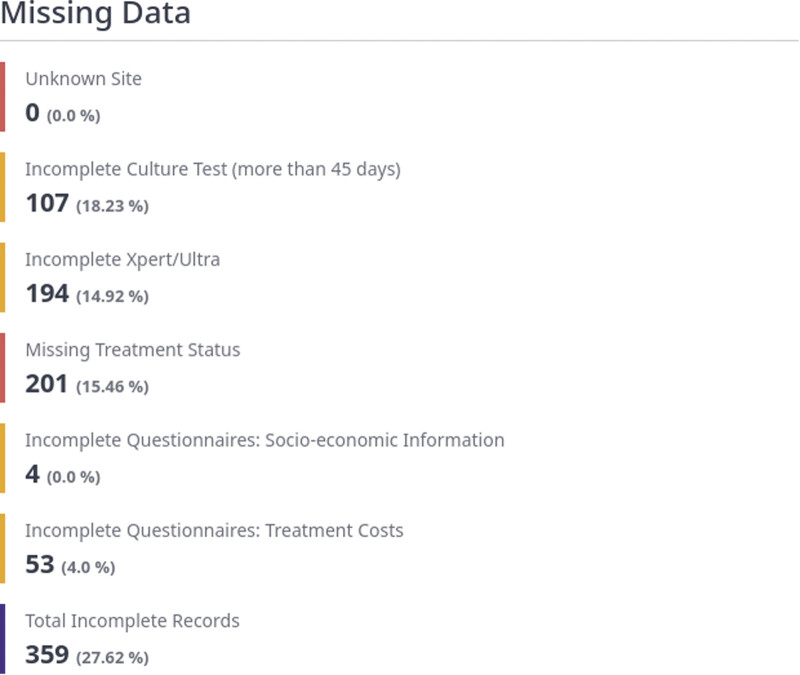
Overview of the total number of missing data. Summarizes the counts of missing values for key inputs, indicating where additional attention is needed.

This QC report also provides a section with totals of missing values. For each relevant input measured, we count how many participants do not have that particular value. Any count other than zero means that participants need additional attention because the required data are missing. Fig. 3 illustrates how missing data are shown. When missing data are entered later (e.g., via offline sync), the report updates automatically on the next hourly run.

#### Incomplete Records Table

**Fig. 4:**
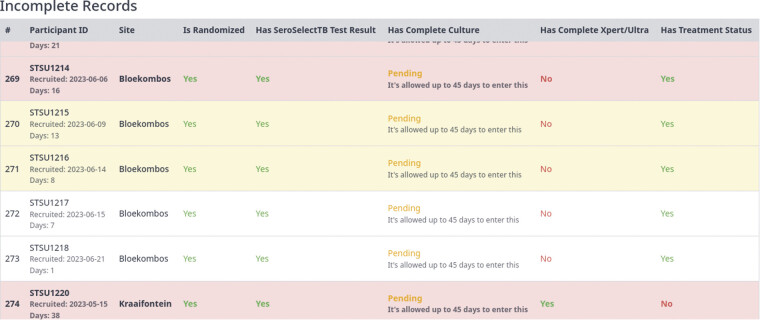
Detailed view of incomplete records by participant ID (PIDs) and site. Lists PIDs and corresponding sites with incomplete data, enabling targeted follow-up.

Based on the statistics presented in Fig. 3 , healthcare workers can utilize the “Incomplete Records” table to identify the participants requiring further attention, as shown in Fig. 4 . This table displays the specific participant IDs (PIDs) and corresponding sites associated with incomplete records, thereby providing a more detailed breakdown of the data summarized in Fig. 3 .

As the system updates, these values will also be updated after the corrections have been entered.

### Online and Offline Mobile Randomization App

To enable uninterrupted participant randomization to study arms in remote sites where electricity and internet access were not guaranteed, the data management team developed a mobile randomization app that could work offline. The app, built using React Native, 9 provides a user-friendly and efficient solution for randomizing participants to study arms. It uses a central synchronization server, built using NodeJS, 10 which stores all the randomized data. Each study site is assigned a separate, isolated account within the app to ensure data security and integrity, with pregenerated randomization blocks stored locally on the device. For security, the randomization data stored locally is encrypted (using device-level secure storage to protect against unauthorized access), and synced over HTTPS to the server; integrity is maintained via unique site accounts and conflict checks during sync, where the server verifies and resolves any discrepancies based on timestamps. The server maintenance cost was minimal (approximately $10/month on the cloud-computing platform Amazon Web Services), making it sustainable for low-resource trials.

#### Randomizing a Participant

**Fig. 5:**
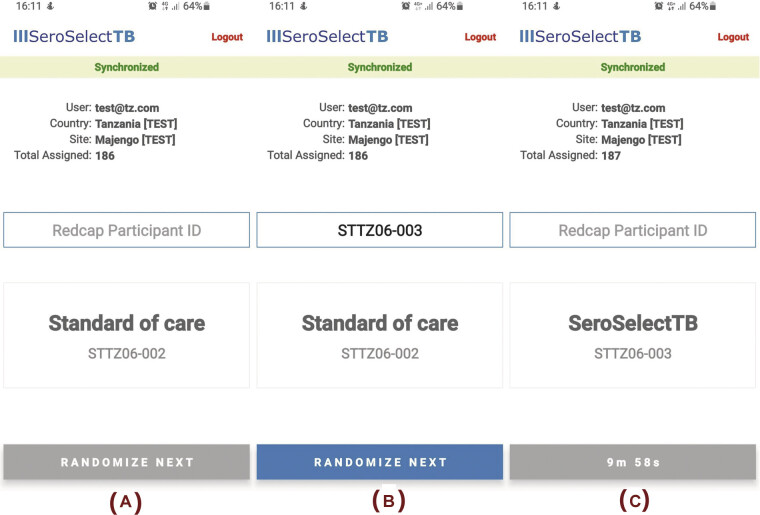
Randomizing a participant to a trial arm. Screenshots from the randomization app show ( **A** ) the last randomized participant, ( **B** ) entry of a new participant ID, and ( **C** ) displayed assignment with a 10-minute randomization lock.

The randomization process within the app follows a block-randomization algorithm to maintain the balance between the study arms, with block sizes of four, six, and eight participants. The block sizes were also randomized to enhance unpredictability and prevent healthcare workers from anticipating assignments, reducing potential bias. This algorithm ensures that both arms are distributed closely at a 1-to-1 ratio, thus minimizing the potential for bias and promoting unbiased participant allocation. The app's interface is simple and intuitive, requiring minimal training for healthcare workers to effectively randomize participants. The randomization steps are shown in Fig. 5 . Once logged in, the healthcare worker sees the last randomized participant ( Fig. 5A ). To randomize a new participant, they enter a new PID in the input field and click the “Randomize Next” button ( Fig. 5B ). After randomizing, the assigned study arm is shown with the entered PID below the arm designation ( Fig. 5C ). PIDs are manually entered by health workers but include site prefixes (e.g., site code) to denote origin; duplicates are prevented by the app, and again during sync. The server resolves any rare conflicts from multi-device use (though sites followed a one-device rule to avoid this).

Fig. 5 illustrates how the app works when an internet connection is available during randomization. Randomizing a participant did not produce a “Not Synchronized” message because synchronization to the server was done immediately.

The randomization app prohibits users from randomizing another participant for the next 10 minutes ( Fig. 5C ). This prevents human errors, for example, randomizing twice, but also prevents healthcare workers from “prerandomizing” and thereby knowing the sequence of the following participants' arm assignments.

Additionally, the randomization app provides a valuable cross-verification mechanism. By comparing the randomized arm displayed in the app with the recorded arm in the eCRF, healthcare workers can identify discrepancies or errors that may have occurred during data entry. This feature enhances the overall data quality and allows for prompt correction of any inconsistencies, ensuring that the recorded arm accurately reflects the randomized assignment for each participant.

#### Automatic Data Synchronization

**Fig. 6:**
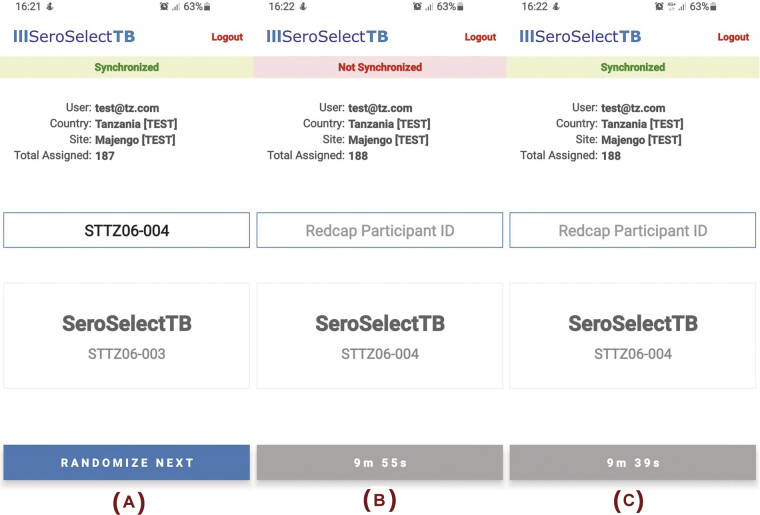
Randomizing while offline and synchronizing when online. Depicts randomization without an internet connection ( **A** ), unsynchronized state ( **B** ), and subsequent synchronization when the connection is restored ( **C** ).

If the healthcare worker randomizes a participant when there is no internet connection, the app will provide the randomized value based on locally stored blocks and store it on the device. When internet access becomes available, the app synchronizes all changes with the central server. This synchronization process ensures that the participant allocation data are securely and accurately recorded, maintaining consistency across all study sites by merging local changes with server data using timestamp-based conflict resolution. Fig. 6A illustrates randomization when there is no internet connection. In Fig. 6B , the “Not Synchronized” message indicates that the app randomized the participant, but that it has not synced the randomization with the server. The app can stay in this state and enable randomization indefinitely. Once an internet connection is established, the app will synchronize all changes from the last synchronization, as shown in Fig. 6C .

### Integration and Customization

Both the QC report and the mobile randomization app can be integrated with any EDC system. For the QC report, the integration can be done quickly when a system provides a way to extract a snapshot of the stored data. The mobile randomization app is independent and can be deployed in parallel with any system. Both systems are also easily customizable and can be adapted to specific use cases for other clinical trials.

## Results

The introduction of an automated QC reporting system significantly improved the efficiency of the SeroSelectTB clinical trial monitoring. Previously, assessing the current state of a clinical trial at any designated time could take months due to manual data aggregation and review processes that required collecting and verifying information from multiple sites, often leading to delays in identifying anomalies. With the automated QC report, this assessment was reduced to just 1 hour, allowing researchers and healthcare workers to obtain near real-time insights into trial progress through dashboards that refreshed automatically. Notably, this process was entirely automated, requiring no manual intervention. Healthcare workers could open the report at any time to access the latest data, eliminating the need for time-consuming data compilation or verification. The experience was uniform across Ethiopia, Tanzania, and South Africa, with no site-specific variations reported. Data summaries extracted from the automated QC report were presented at monthly steering committee meetings throughout the SeroSelectTB study, providing factual updates on enrollment and quality metrics that advanced study coordination.

Frequent updates to the QC report also improved trial oversight by allowing healthcare workers to promptly detect and correct errors, such as missing values or inconsistencies. In many cases, corrections could be made while the participant was still present, reducing the risk of incomplete or inaccurate data entry and minimizing follow-up efforts. This immediate feedback mechanism contributed to higher data quality and ensured protocol adherence by flagging deviations early. The report also acted as a structured reminder, prompting healthcare workers to complete necessary follow-up actions after predefined time intervals. This feature reduced the likelihood of missed procedures, improving overall trial compliance and data completeness.

Additionally, the QC report provided both high-level summaries and granular details to keep all stakeholders informed. High-level insights helped investigators monitor trends and detect patterns, while detailed data allowed healthcare workers to track individual participants precisely. By presenting comprehensive information in a user-friendly format, the report supported effective decisions and enhanced trial management.

The mobile randomization app allowed healthcare workers to continue tasks even in areas with limited or no internet access. This capability was particularly valuable in remote or resource-constrained settings, where stable internet connectivity was not always available, allowing uninterrupted operations during outages. As a result, more participants were successfully enrolled in the clinical trial, reducing delays and ensuring a steady recruitment rate. Notably, offline access contributed to higher weekly recruitments at affected sites. By eliminating reliance on constant internet access, the application enhanced operational efficiency and expanded trial accessibility to a broader population.

The block-randomization algorithm ensured balanced allocation between study arms, minimizing selection bias and maintaining data integrity. This close balance reinforced the validity of results, while the automated process eliminated human influence, ensuring objective and reproducible participant assignment. No statistically significant differences in baseline characteristics were observed, as no manual phase was used; all randomization was digital from the start.

By eliminating subjective decision-making, the system upheld the methodological rigor essential for high-quality clinical research.

## Discussion

The implementation of an automated QC reporting tool and an offline participant randomization app demonstrates how targeted digital solutions can address structural weaknesses in decentralized clinical trials, particularly in low-resource environments. Rather than replacing existing EDC systems, these tools functioned as complementary layers, extending oversight and operational continuity where conventional systems assume stable infrastructure.

### Effect of the Automated QC Reporting Tool

The automated QC reporting tool shifted monitoring from retrospective review to near real-time surveillance. Traditional trial oversight often relies on periodic monitoring visits or manual aggregation of site data. In contrast, hourly automated extraction from REDCap enabled earlier detection of missing data, duplicate entries, and logical inconsistencies.

Beyond error detection, the system enabled cross-site comparisons and trend visualization, supporting remote supervisory oversight. This model resembles risk-based monitoring frameworks increasingly adopted in global trials, but implemented in a lightweight, resource-efficient manner.

The tool's reminder function proved particularly useful for long-latency endpoints such as mycobacterial culture results. In resource-limited contexts, delayed laboratory feedback often contributes to silent data gaps; automated flagging reduces this risk.

A limitation is that the QC system depends on accurate source entry in REDCap. Automation improves detection speed but does not replace source-data verification or clinical oversight.

In Tanzania, the QC report was reported as instrumental in tracking daily enrollment and comparing study data with the REDCap system. It flagged missing data, prompting research assistants to follow up and fill in incomplete records. The ability to communicate issues daily enhanced coordination between sites and improved data accuracy.

Beyond flagging missing data, in South Africa, the QC report also served a valuable social function between the healthcare workers and the scientists or project managers. The report facilitated discussions addressing overall data quality and missing data flags, including laboratory results, in particular culture results. Mycobacterial culture can take up to 6 weeks before results are available, and may thus be overlooked or forgotten without proactive follow-up measures, but the tool's reminders ensured timely checks.

### Effect of the Offline Randomization App

Healthcare workers reported that the offline randomization app provided substantial benefits, particularly in remote areas with limited internet connectivity, where traditional web-based systems would fail during outages. The ability to enroll participants immediately reduced delays and ensured compliance with randomization protocols by allowing assignments even without power or network access. In South Africa, the team noted that the app's intuitive interface required minimal training, allowing healthcare workers to integrate it seamlessly into routine diagnostic workflows without disrupting patient care.

A key advantage of the app was to maintain strict allocation sequences, preventing bias or deviations from the protocol through its block-based algorithm and 10-minute lockout feature. In Ethiopia, healthcare workers emphasized that the app ensured assignment fairness and helped avoid any perceived favoritism, while the built-in 10-minute interval between randomizations allowed adequate time for documentation, further reducing the risk of misclassification. This built-in delay helped prevent anticipatory assignment or rapid repeats, reinforcing the integrity of the randomization process in South Africa, where sites had high numbers of patients.

Compared with previous trials relying on web-based systems, paper-based randomization lists, or physical monitoring visits by appointed experts, 11 12 this app offered a more reliable and consistent approach. Healthcare workers in Tanzania noted that it eliminated favoritism and assumptions that were sometimes present in manual randomization methods. The digital record of randomized participants facilitated transparency, ease of verification, and compliance with study requirements, with no instances of favoritism observed in this digital-only trial.

### Challenges Addressed by the Offline Randomization App

Network dependency remains a common vulnerability in multinational trials conducted in HBC settings. The offline-first architecture directly addressed this limitation by allowing secure local assignment with later synchronization.

Importantly, the app also reduced behavioral bias. In settings where manual or discretionary assignment previously occurred, the enforced digital workflow removed subjective influence. This aligns with broader trends toward automation as a mechanism for safeguarding methodological rigor, particularly in pragmatic trials conducted outside tertiary research centers.

Initial resistance from some healthcare workers reflected a transition from discretionary to protocol-enforced allocation. However, strict adherence improved allocation equity and transparency.

### Broader Implications

The integration of these tools illustrates a practical principle: infrastructure-adaptive design may be more effective than feature-rich centralized systems in resource-constrained trials.

Strengths include low implementation cost, scalability across countries, adaptability to existing EDC systems, and uniform adoption across sites. Limitations include dependence on correct device management practices and the absence of a formal before/after randomized evaluation of efficiency gains.

Nevertheless, quantifiable improvements in monitoring speed and enrollment continuity suggest meaningful operational benefit. Future refinement could include: enhanced anomaly detection algorithms, expanded offline functionality beyond randomization, and integration with risk-based monitoring dashboards.

The feedback from healthcare workers underscores the necessity of such tools in future trials. These tools can be extrapolated to other uses, such as outbreak monitoring or non-TB trials, improving data acquisition in resource-poor areas. The project teams reported that the digital tools introduced improvements that were transformative for trials in low-resource settings, minimizing administrative burden and errors with minimal additional infrastructure. Documenting these gains can facilitate the use of digital methods in similar trials, promoting wider adoption.

## Conclusion

This study demonstrates that purpose-built digital extensions to conventional EDC systems can materially strengthen trial oversight and continuity in infrastructure-variable environments.

The mobile randomization application ensured balanced and unbiased allocation while maintaining enrollment during connectivity disruptions. The automated QC reporting system transformed monitoring from delayed review to near real-time supervision, reducing error detection time from weeks to hours.

The benefits of implementing IT solutions are substantial. The effect of these tools extends beyond operational challenges in clinical trials to broader research implications. Together, these tools improved protocol adherence, reduced operational delay, enhanced data completeness, and maintained methodological rigor.

Rather than proposing a universal platform, this work supports a modular, adaptable approach to digital trial management, where targeted automation addresses specific operational vulnerabilities.

As clinical trials expand into diverse and decentralized environments, infrastructure-resilient design, lightweight automation, and interoperability with existing systems will become increasingly important. Continued development of scalable, low-cost digital oversight tools may improve research quality not only in TB studies, but across global health research more broadly.

## Notes

**Clinical Relevance Statement**
Automated QC reporting and offline randomization improve data reliability and trial efficiency in resource-limited environments. These methods enable real-time monitoring and unbiased participant assignment, and provide a sustainable approach for digital trial management.

## Notes

**Multiple-Choice Questions**

1. What is a major benefit of an automated QC report in clinical trials?
  - Faster participant recruitment.
  - Early detection of data errors.
  - Improved patient satisfaction.
  - Reduced randomization bias.
  **Correct Answer:** The correct answer is option b. Early detection of data errors. The QC report enables early detection of data quality issues.
2. What was the main advantage of the offline randomization app in the SeroSelectTB trial?
  - Reduced trial cost.
  - Enabled randomization without internet access.
  - Simplified regulatory approval.
  - Increased test accuracy.
  **Correct Answer:** The correct answer is option b. Enabled randomization without internet access. The app worked offline, ensuring unbiased participant allocation in remote areas.
